# A multimodal fusion method for Alzheimer’s disease based on DCT convolutional sparse representation

**DOI:** 10.3389/fnins.2022.1100812

**Published:** 2023-01-06

**Authors:** Guo Zhang, Xixi Nie, Bangtao Liu, Hong Yuan, Jin Li, Weiwei Sun, Shixin Huang

**Affiliations:** ^1^School of Communication and Information Engineering, Chongqing University of Posts and Telecommunications, Chongqing, China; ^2^School of Medical Information and Engineering, Southwest Medical University, Luzhou, China; ^3^Chongqing Key Laboratory of Image Cognition, College of Computer Science and Technology, Chongqing University of Posts and Telecommunications, Chongqing, China; ^4^School of Optoelectronic Engineering, Chongqing University of Posts and Telecommunications, Chongqing, China; ^5^Department of Scientific Research, The People’s Hospital of Yubei District of Chongqing City, Yubei, China

**Keywords:** Alzheimer, multimodal, sparse, fusion, convolutional

## Abstract

**Introduction:**

The medical information contained in magnetic resonance imaging (MRI) and positron emission tomography (PET) has driven the development of intelligent diagnosis of Alzheimer’s disease (AD) and multimodal medical imaging. To solve the problems of severe energy loss, low contrast of fused images and spatial inconsistency in the traditional multimodal medical image fusion methods based on sparse representation. A multimodal fusion algorithm for Alzheimer’ s disease based on the discrete cosine transform (DCT) convolutional sparse representation is proposed.

**Methods:**

The algorithm first performs a multi-scale DCT decomposition of the source medical images and uses the sub-images of different scales as training images, respectively. Different sparse coefficients are obtained by optimally solving the sub-dictionaries at different scales using alternating directional multiplication method (ADMM). Secondly, the coefficients of high-frequency and low-frequency subimages are inverse DCTed using an improved L1 parametric rule combined with improved spatial frequency novel sum-modified SF (NMSF) to obtain the final fused images.

**Results and discussion:**

Through extensive experimental results, we show that our proposed method has good performance in contrast enhancement, texture and contour information retention.

## 1. Introduction

Alzheimer’s disease (AD) is a common neurodegenerative disease with concealed onset and incurable in the elderly. In clinical, AD is characterized by general dementia such as cognitive decline and memory loss (Dubois et al., 2021). Advanced multimodal neuroimaging techniques, such as magnetic resonance imaging (MRI) (Thung et al., 2014; Liu M. et al., 2016; Lian et al., 2018; Fan et al., 2019) and positron emission tomography (PET) (Chetelat et al., 2003; Liu M. et al., 2017) use different imaging mechanisms to reflect the location of organs or lesions from different angles, and can clearly show human tissue or metabolism and blood flow of organs. This technique has the complementarity and irreplaceability of medical information, which provides a good prospect for the early diagnosis of AD (Perrin et al., 2009; Mohammadi-Nejad et al., 2017; Wang et al., 2021).

Medical image fusion includes image decomposition, fusion rules, and image reconstruction. The main purpose of image decomposition is to extract the feature information from the image. The effectiveness of feature extraction determines the quality of fusion results. The current image fusion algorithms can be divided into three categories. The first kind of image fusion is based on wavelet and pyramid transform (Da Cunha et al., 2006; Yang et al., 2010; Miao et al., 2011; Liu S. et al., 2017; Liu X. et al., 2017). Among them, the Laplace pyramid transform has the best robustness in the sampling operator. Wang and Shang (2020) proposed a fast image fusion method based on discrete cosine transform (DCT), which decomposes each source image into a base layer and a detail layer for image fusion. And optimize the calculation method of the base layer to better preserve the structure of the image. In addition, non-subsampled shear wave transforms (NSST) (Kong et al., 2014) are also widely used in AD diagnosis because of their translation invariance and multidirectional. The second kind of image fusion is based on edge-preserving filtering (Farbman et al., 2008; Xu et al., 2011; He et al., 2012; Hu and Li, 2012; Zhang et al., 2014; Kou et al., 2015). This method can filter the image while erasing the details and retaining its strong edge structure. It can decompose the input image into smooth layers and detail layers. The smooth layer contains the main energy information of the image; the detail layer contains texture features. The third type is the feature selection method based on sparse learning, for example, the multiplier alternating directional multiplication method (ADMM) algorithm (Liu and Yan, 2011) organizes the whole learning and decomposition process into vectors, and iterates with a sliding window to achieve the convergence effect.

Sparse representation (SR) is a widely used image representation theory. It deals with the natural sparsity of signals according to the physiological characteristics of the human visual system. It is widely used in image classification (He et al., 2019), image recognition (Liu H. et al., 2016), image feature extraction (Liu et al., 2014), and multimodal image fusion (Zhu et al., 2016). The fusion method based on SR and dictionary learning is widely used in image fusion proposed by compressed sensing theory (Donoho, 2006), and it is generally better than most traditional fusion methods (Zhang and Patel, 2017). It usually represents the source image in the form of a linear combination of overcomplete dictionaries and sparse coefficients. Because the weighted coefficients obtained are sparse, the significant information of the source image can be represented by a small number of non-zero elements in the sparse coefficients. In the methods based on SR, sparse coding is usually based on local image blocks. Yang and Li (2009) first introduces SR into image fusion. This method uses sliding window technology to make the fusion process robust to noise and registration errors. Because the adjacent image blocks overlap each other, the result of each pixel is multi-valued. Ideally, multiple values of each pixel should be equal to maintain the consistency of overlapping image blocks (Gu et al., 2015). However, sparse coding is performed independently on each image block. The correlation between image pixels is ignored, resulting in multiple unequal values for each pixel. At the same time, most fusion methods adopt the strategy of aggregation and averaging to obtain the final value of each pixel, which will cause the image details to be smoothed or even lost in fusion (Rong et al., 2017). Yin et al. (2016) obtained a joint dictionary by using the source image as training data and then fused the image using the maximum weighted multi-norm fusion rule. But the problem of missing details still exists. Zong and Qiu (2017) proposed a fusion method based on classified image blocks, which uses directional gradient histogram (HOG) features to classify image blocks to establish a sub-dictionary. Although the problem of loss of details has been reduced, it still inevitably leads to some details being smoothed. Zhang and Levine (2016) proposed an improved fusion method of multitasking robust SR combined with spatial context information. Like most methods based on SR, this method encodes for local image blocks rather than for the whole image. As a result, it can still lead to poor preservation of details. And usually, the fusion methods based on sparse coding use only one dictionary to represent the different morphological parts of the source image, which is easy to cause the loss of image information.

Therefore, we propose a multimodal fusion method for Alzheimer’s disease based on DCT convolution SR to solve the above problems. It was evaluated on the neuroimaging database of Alzheimer’s disease (ADNI) (Veitch et al., 2022), and its effectiveness was verified by experiments.

The contribution of this paper has the following three aspects:

1. An improved multiscale decomposition method of DCT is proposed. Firstly, the M × N size image is divided into blocks of 8 × 8 size, and then the DCT transform is applied to each small block separately. The DCT coefficients of each image block are normalized separately and their low-order DCT coefficients are calculated. The ratio of the energy of the higher-order DCT coefficients to the energy of the lower-order DCT coefficients is used as the focus evaluation function. To solve the problem of fused image capability loss and contrast reduction.

2. A convolutional SR method is proposed to solve the problem of spatial inconsistency of multimodal image fusion by combining the high-frequency and low-frequency components obtained from the multiscale decomposition and adopting the improved rules of spatial frequency and L_1_ parametric combination according to the characteristics of AD multimodal images.

3. To address the problem of limited detail preservation capability of medical image fusion methods based on SR and the lack of expression capability of single dictionary, the detail texture and contour of the fused image are enhanced by constructing multiple sub-dictionaries, and finally the fused detail layer image is fused and reconstructed with the fused base layer image to obtain the fused image. The fused AD medical information features are preserved.

## 2. Materials and methods

**FIGURE 1:**
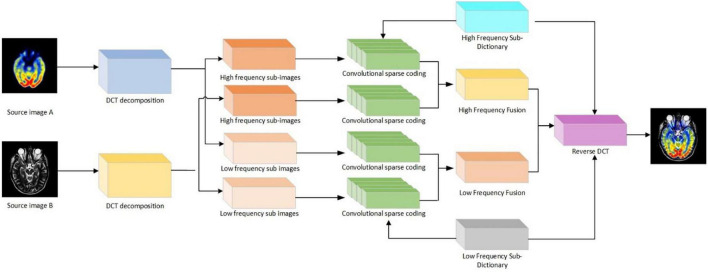
Flowchart of image fusion algorithm for discrete cosine transform (DCT) transform.

In the process of image fusion, the most important thing is how to extract low-high-frequency coefficients and the fusion criteria of low-high-frequency coefficients. First of all, the DCT transform is used to decompose the MRI image in multi-scale; the DCT coefficients of each image block are normalized respectively, and its low-order DCT coefficients are calculated. The ratio of the energy of the higher-order DCT coefficient to that of the lower-order DCT coefficient is used as the focusing evaluation function. Then, the sub-images on each scale are convoluted sparsely encoded, and the sparse coefficients of different sub-images are obtained. The high-frequency sub-image coefficients are combined with the improved L_1_ norm and the novel sum-modified SF (NMSF), and the low-frequency sub-images are fused with the improved L_1_ norm and regional energy. Finally, the fused low-frequency sub-band and high-frequency sub-band are transformed by inverse DCT transform to get the final fused image. The principle of the image fusion algorithm based on DCT transform is shown in Figure 1:

### 2.1. DCT decomposition

#### 2.1.1. Low-frequency component

The most important part of information for vision is concentrated in the low frequencies of the image. Low frequencies represent the slow variation between image pixels. It is the large flat area of the image that describes the main part of the image and is a comprehensive measure of the intensity of the whole image. In order to maintain the visibility of the image, the low-frequency part of the image is preserved, and changes in the low-frequency part may cause large changes in the image. The low-frequency coefficients of the fused image based on the DCT transform are averaged, assuming that there are *p* multi-exposure images, which can be defined as:


(1) $$G\,\left(i,j\right)=\sum_{k=1}^{p}w_{k}\,G_{k}\,\left(i,j\right)$$

(2) $$\sum_{k=1}^{p}w_{k}=1$$

(3) $$w_{1}=w_{2}=\cdots =w_{n}=\cdots \,w_{p}=1/p$$

where *G*_*k*_(*i*,*j*) is the low-frequency coefficients extracted from the source image after DCT transformation; *G*(*i*,*j*) is the fused low-frequency coefficients; and *w*_*k*_ is the weighting factor.

#### 2.1.2. High-frequency component

The high-frequency coefficients correspond to detailed information in the image, such as edges, and are extracted from the 8 × 8 chunked image after the DCT transform. The standard deviation of the high-frequency coefficients *D*(*i*,*j*) in the (2*k* + 1)×(2*k* + 1) neighborhood centered on pixel (*i*,*j*) is calculated separately.


(4) $$C\,\left(i,j\right)=\sqrt{\frac{\sum_{m=i-k}^{i+k}\sum_{n=j-k}^{j+k}{\left[D\,\left(m,n\right)-\bar{M}\,\left(m,n\right)\right]}^{2}}{d-1}}$$

where, *D* is the number of pixel points in the region (2*k* + 1)×(2*k* + 1); *D* is the value of the high frequency coefficient corresponding to the (*m*,*n*) point; $\bar{M}\,\left(m,n\right)$ is the average value of pixels in the region, which can be defined as:


(5) $$\bar{M}\,\left(i,j\right)=\frac{1}{d}\,\sum_{m=i-k}^{i+k}\sum_{n-j-k}^{j+k}D\,\left(m,n\right)$$

The regional standard deviation of the high-frequency coefficients for each of the *P* multi-exposure images is [*C*1(*i*,*j*),*C*2(*i*,*j*),…,*Cp*(*i*,*j*)], then the weight coefficients corresponding of the extracted high-frequency coefficient is:


(6) $$w_{k}\,\left(i,j\right)=\frac{{\text{C}}_{k}\,\left(i,j\right)}{\sum_{k=1}^{p}{\text{C}}_{k}\,\left(i,j\right)}$$

where, the weights of the *P* multi-exposure images are compared to them, the fused high-frequency coefficient *D*(*i*,*j*) is the high-frequency coefficient corresponding to the largest weighting factor.


(7) $$w_{k}\,\left(i,j\right)=\max\left[w_{1}\,\left(i,j\right),w_{2}\,\left(i,j\right),\cdots ,w_{k}\,\left(i,j\right),\cdots ,w_{p}\,\left(i,j\right)\right]$$

(8) $$D\,\left(i,\,j\right)=D_{k}\,\left(i,\,j\right)$$

### 2.2. Sparse representation

The medical image fusion method consists of the following four parts: (1) Multi-scale dictionary learning to train sub-dictionaries on different scales of sub-images as training images. (2) Convolutional sparse coding of the dictionaries at different scales to find their convolutional sparse coefficients. (3) Low-frequency sub-band coefficient fusion rules for low-frequency sub-images are fused according to the set fusion rules. (4) High-frequency sub-band coefficient fusion rules fuse high-frequency sub-images at different scales.

#### 2.2.1. Multi-scale dictionary learning

The source images A and B are firstly decomposed by *l*-level DCT to obtain their decomposition coefficients $\left\{H_{A}^{l,k},L_{A}\right\}$ and $\left\{H_{B}^{l,k},L_{B}\right\}$, respectively. Where, $H_{A}^{l,k}$ and $H_{B}^{l,k}$ denote the high-frequency sub-band coefficients of source images A and B at decomposition scale *l* and orientation *k*. *L*_*A*_ and *L*_*B*_ are the low-frequency sub-band coefficients of images A and B, respectively. The sub-band images of each scale are used as training images to train the corresponding convolutional sparse sub-dictionaries. The different convolutional sparse sub-dictionaries capture the features of the sub-images at different scales. Finally, the low-frequency and high-frequency sub-dictionaries are formed by combining the sub-dictionaries at different scales. The high-frequency first-scale images $H_{A}^{l,k}$ and $H_{B}^{l,k}$ of source images A and B are used as training images ${\left\{y_{m}\right\}}_{m=1}^{M}$, and the corresponding convolutional sparse dictionary learning models are built. The formula is as follows:


(9) $$\underset{d,x}{\arg\min}\frac{1}{2}\,\sum_{m=1}^{M}{\left\|y_{m}-\sum_{k=1}^{K}d_{k}\times x_{m,k}\right\|}_{2}^{2}+\lambda \,\sum_{m=1}^{M}\sum_{k=1}^{K}{||x_{m,k}||}_{1}$$

where, *x*_*m*,*k*_ is the sparse coefficient corresponding to the *m*th training image; *d*_*k*_ is the corresponding filter; * denotes the two-dimensional convolution operation; λ is the regularization parameter; and ||⋅||_1_ denotes the *l*_1_ parametric number, which represents the sum of the absolute values of the elements.

(1) Dictionary update phase. By keeping the sparse coefficients constant, each filter is optimally updated with the following equation:


(10) $$\underset{d_{k}}{\arg\min}\frac{1}{2}\,\sum_{m=1}^{M}{\left\|y_{m}-\sum_{k=1}^{K}d_{k}\times x_{m,k}\right\|}_{2}^{2}$$

To optimize the filter in the discrete Fourier domain, the filter *d*_*k*_ needs to be zero-filled to the same size as *x*_*m*,*k*_. Taking into account the normalization of *d*_*k*_ with zero padding, the formula is as follows:


(11) $$\underset{\left\{d_{m}\right\},\left\{g_{m}\right\}}{\arg\min}\frac{1}{2}\,\sum_{k}{\left\|\sum_{m}x_{m,k}\times d_{m}-s_{k}\right\|}_{2}^{2}+\sum_{m}l_{C_{P\,N}}\,\left(g_{m}\right)$$

The ADMM algorithm shows that *C*_*PN*_ = {*x* ∈ *R^N^*:(*I*−*PP^T^*)*x* = 0,||*x*||_2_ = 1} represents the constraint range, the indicator function is defined as $l_{s}(X)=\{\begin{array}{cc} 0 & \text{if}\;X\in S\\ \infty & \text{if}\;X\notin \;S \end{array}$, and *g*_*m*_ is an auxiliary variable introduced to facilitate optimal derivation. The resulting updated convolution filter *d*_*k*_ can be obtained.

(2) Convolutional sparse coefficient update phase. Update the coefficients by keeping the filter unchanged:


(12) $$\underset{x_{m,k},z_{m,k}}{\arg\min}\frac{1}{2}\,\sum_{m=1}^{M}{\left\|y_{m}-\sum_{k=1}^{K}d_{k}\times x_{m,k}\right\|}_{2}^{2}+\lambda \,\sum_{m=1}^{M}\sum_{k=1}^{K}{||z_{m,k}||}_{1}$$

where, *z*_*m*,*k*_ is the introduced auxiliary variable. We obtain the updated convolutional sparse coefficients by alternating iterative solutions.

**FIGURE 2:**
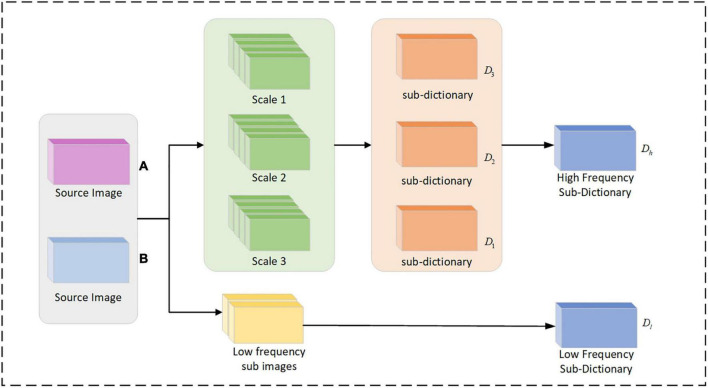
Flow chart of multi-scale dictionary learning.

In Figure 2, the cyclic execution of the dictionary and the convolutional sparse coefficients are updated to a predetermined maximum number of cycles or a set parameter threshold to stop. The convolutional sub-dictionary *D*_1_ for the first scale of the high-frequency sub-image is output. The second and third scales of the high-frequency sub-images are dictionary learned separately to obtain the convolutional sub-dictionaries *D*_2_, *D*_3_. By combining each high-frequency sub-dictionary, and the high-frequency dictionary *D*_*h*_ = [*D*_1_,*D*_2_,*D*_3_] is obtained. The low-frequency sub-images are subjected to dictionary learning, and the low-frequency dictionary *D*_*l*_ is obtained.

#### 2.2.2. Convolutional sparse coding

To better capture the detailed texture information of medical images and reduce the influence of artifacts, a high-frequency dictionary *D*_*h*_ and a low-frequency dictionary *D*_*l*_ are obtained by learning. Convolutional sparse coding is performed on the decomposition coefficients $\left\{H_{A}^{l,k},L_{A}\right\}$ of the source image A. The TV regularization is then incorporated into the convolutional sparse coding model. The formula is as follows:


(13) arg⁡min{xk}12⁢‖s-∑kdk×xk‖22+λ⁢∑k||xk||1+λ1⁢T⁢V⁢(∑k=1Kdk⁢xk*)

where, *TV*(*X*) = ||*g*_0_×*x*||_1_ + ||*g*_0_×*x*||_1_, *g*_0_ and *g*_1_ are the filters used to calculate the gradients along the rows and columns of the image, respectively. The sparsity coefficients $\left\{x_{m,L}^{A},x_{m,l,k,H}^{A}\right\}$ and $\left\{x_{m,L}^{B},x_{m,l,k,H}^{B}\right\}$ of the coefficients in the sub-bands of the source images A and B, respectively, are obtained by optimal solution in the discrete Fourier domain. Where, *m* denotes the number of filters and convolutional sparse coefficient maps; *L* denotes the low frequency image; *H*denotes the high frequency image; and *l* and *k* denote the scale and orientation of the corresponding sub-bands, respectively.

#### 2.2.3. Low-frequency coefficient fusion rules

After DCT decomposition, the energy information of the image is contained in the low-frequency sub-bands *L*_*A*_ and *L*_*B*_ of the source images A and B, which are displayed as basic information such as the contour and brightness of the image. The averaging strategy generally used for low-frequency coefficient fusion tends to lead to a reduction in the contrast of the image. In the case of fusion using the *Max*−*L*_1_ rule with SR, the reduction of contrast can be effectively avoided. However, it can lead to the problem of spatial inconsistency of the image. At the same time, because the region energy can better reflect the energy and significant features of the image, and the convolution sparse coefficients of the *L*_1_ parameter averaging strategy can effectively reduce the effect of misalignment. Therefore, a combination of region energy and averaged *L*_1_ parameter is used to fuse the low-frequency sparse coefficients.


(14) $$L_{F}=\sum_{m=1}^{M}d_{m}^{l}\times x_{m,L}^{F}$$

#### 2.2.4. High-frequency coefficient fusion rules

The high frequency sub-bands $H_{A}^{l,k}$ and $H_{B}^{l,k}$ of the source images A and B contain a large amount of information such as texture details of the images. The convolutional SR of the fusion method has good performance in preserving detail information, and the improved spatial frequency and can well reflect the gradient changes of the image texture. Therefore, the improved spatial frequency combined with the average *L*_1_ parameter strategy is used to fuse the high frequency sparse coefficients.


(15) $$H_{F}^{l,k}=\sum_{m=1}^{M}d_{m}^{h}\times x_{m,l,k,H}^{F}$$

#### 2.2.5. Multimodal medical image fusion

The problem of spatial inconsistency in multimodal images is caused when the *L*_1_ parametric maximum fusion rule is used in traditional SR-based fusion methods. Therefore, we decompose the source image by performing DCT on it. Different sub-dictionaries are trained for features of different scales. A rule combining region energy and activity coefficients is used for fusion of the low frequency component coefficients, and a modified rule combining spatial frequency and activity coefficients is used for fusion of the high frequency component coefficients. The problems of reduced contrast, blurred details and inadequate information extraction are avoided. The specific steps are as follows:

(1) The DCT decomposition of source images A and B is performed to obtain the respective decomposition coefficients $\left\{H_{A}^{l,k},L_{A}\right\}$ and $\left\{H_{B}^{l,k},L_{B}\right\}$.

(2) In the dictionary learning stage, the images at different scales corresponding to the multimodal source images are used as training sets, and the sub-dictionaries *D*_0_, *D*_1_, *D*_2_, *D*_3_ corresponding of each scale is derived. The low-frequency dictionary is *D*_*l*_ = *D*_0_. The high-frequency dictionary is *D*_*h*_ = [*D*_1_,*D*_2_,*D*_3_].

(3) Sparse coding stage. Convolutional sparse coding is performed on the sub-images of different orientations at each scale to obtain the corresponding convolutional sparse $\left\{x_{m,L}^{A},x_{m,l,k,H}^{A}\right\}$ and $\left\{x_{m,L}^{B},x_{m,l,k,H}^{B}\right\}$.

(4) Low-frequency component fusion stage. The regional energies *E*_*A*_ and *E*_*B*_ of *L*_*A*_ and *L*_*B*_, and the active level maps ${\bar{\alpha }}_{A}$ and ${\bar{\alpha }}_{B}$ are calculated. The convolution sparsity coefficients $x_{m,L}^{F}$ are obtained after fusion. Finally, the convolution sparse coefficients are reconstructed with the low-frequency dictionary convolution to obtain the low-frequency sub-band image *L*_*F*_.

(5) High-frequency component fusion stage. The fused convolutional sparse coefficients C are obtained by calculating the improved spatial frequencies of $H_{A}^{l,k}$ and $H_{B}^{l,k}$. Then the high-frequency sub-band images $H_{F}^{l,k}$ are obtained by convolutional fusion with the high-frequency dictionary *D*_*h*_.

(6) Finally, the fused image F is obtained by performing inverse DCT on the fused sub-band image {HF,′k,LF}.

## 3. Results and discussion

### 3.1. Data set and training parameter settings

(1) Experimental settings

All our experiments are conducted on a computer with Intel Core i7-10750H CPU 2.60 GHz, 16 GB RAM, NVIDIA GeForce GTX 3090 Ti. We train the convolutional sparse and low-rank dictionary with sliding step size set to 1, sliding window size set to 8×8, dictionary size set to 64×512, error set to *ℰ* = 0.03, and decomposition level set to 3.

(2) Data sets and comparison methods

To validate the performance of the proposed method. We selected 136 sets of aligned AD brain medical images (image size of 256×256 pixels) as the source images to be fused. All image slices were obtained from the Harvard Whole Brain Atlas database (Johnson and Becker, 2001), and the three AD medical image types included 42 sets of CT-MRI images; 42 sets of MRI-PET images; and 52 sets of MRI-SPECT images. Four contrast algorithms were adopted for comparison, including nonsubsampled contourlet (NSCT) (Li and Wang, 2011), NSST (Kong et al., 2014), guided filter ng fusion (GFF) (Li et al., 2013), and Laplacian redecomposition (ReLP) (Li et al., 2020).

(3) Objective evaluation metrics

We selected 10 metrics for objective index evaluation and analysis: mutual information (MI) (Xydeas and Petrovic, 2000), natural image quality evaluator (NIQE) (Mittal et al., 2012), average gradient (AG) (Du et al., 2017), edge intensity (EI) (Wang et al., 2012), tone-mapped image quality index (TMQI) (Yeganeh and Wang, 2012), spatial frequency (SF) (Eskicioglu and Fisher, 1995), SD (Liu et al., 2011), root mean square error (RMSE) (Zhang et al., 2018), gradient similarity mechanism (GSM) (Liu et al., 2011), and VIF (Sheikh and Bovik, 2006). SF is the spatial frequency, which is a measure of the richness of image detail and reflects the sharpness of image detail. A larger value means that the image detail is richer. SD is the standard deviation, which measures the contrast of the image; a larger value indicates a better contrast of the image. RMSE is the root mean square error, which measures the difference between the fused image and the source image; a smaller value indicates that the fused image information is closer to the source image. GSM measures the gradient similarity between images; a larger value indicates that the gradient information of the fused image is closer to the source image. NIQE index, the smaller the value, the smaller the distortion. VIF is an image information measure that quantifies the information present in the fused image; larger values indicate better fusion. NIQE measures the simple distance between the model statistic and the distorted image statistic. AG indicates the average gradient, which is used to extract the contrast and texture change features of the image. EI reflects the sharpness of the edges. TMQI index measures the significant features of brightness and contrast between the reference image and the fused image, and measures the structural fidelity of the fused image. Larger values of MI, SF, AG, EI, and TMQI indexes indicate better fusion.

### 3.2. CT-MRI fusion results comparison

**FIGURE 3:**
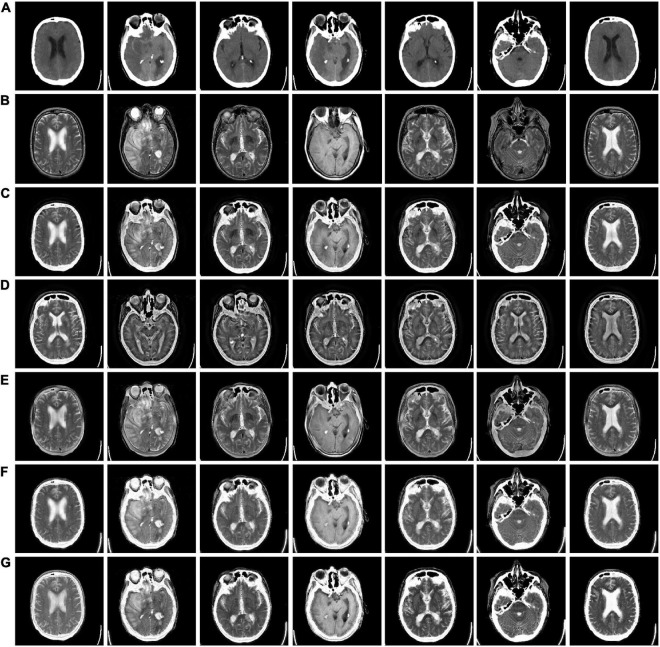
CT-MRI fusion images obtained by five fusion methods. **(A)** Computed tomography (CT) source image; **(B)** magnetic resonance imaging (MRI) source image; **(C)** nonsubsampled contourlet (NSCT) fusion result; **(D)** non-subsampled shear wave transform (NSST) fusion result; **(E)** guided filter ng fusion (GFF) fusion result; **(F)** Laplacian redecomposition (ReLP) fusion result; **(G)** fusion result of the proposed method.

In Figure 3, we used 42 sets of CT-MRI fused images and randomly selected seven fused images for comparison. From the figure, we can see that the images fused by NSCT and GFF algorithms are too dark. The images fused by NSST are not only darker but also distorted. The images fused by ReLP algorithm have better brightness but not enough texture details. Our fusion algorithm performs best in terms of brightness, detail texture and edge contour.

The mean values of objective evaluation metrics for fusion results corresponding to different rules are given in Table 1, where bold indicates that the method ranks best in the metrics. NSCT is low in SD in terms of metrics, indicating insufficient image contrast. NSST is lowest in SF in terms of metrics, with poor image details. ReLP is less distorted with our method in terms of RMSE and GSM. Our proposed method performs better performance in terms of color retention, contrast, and detail retention, and achieves the optimum.

**TABLE 1:** Average values of index evaluation of different fusion methods for CT-MRI.

Methods	SF	SD	RMSE	GSM	VIF
NSCT	23.5217	1.4275	0.1617	0.9631	0.2376
NSST	23.3269	1.4729	0.1678	0.9620	0.2582
GFF	23.8825	1.4681	0.1604	0.9715	0.2265
ReLP	24.2733	1.4953	0.1539	0.9743	0.2674
Proposed	25.1246	1.5203	0.1482	0.9776	0.2743

### 3.3. MRI-PET fusion results comparison

**FIGURE 4:**
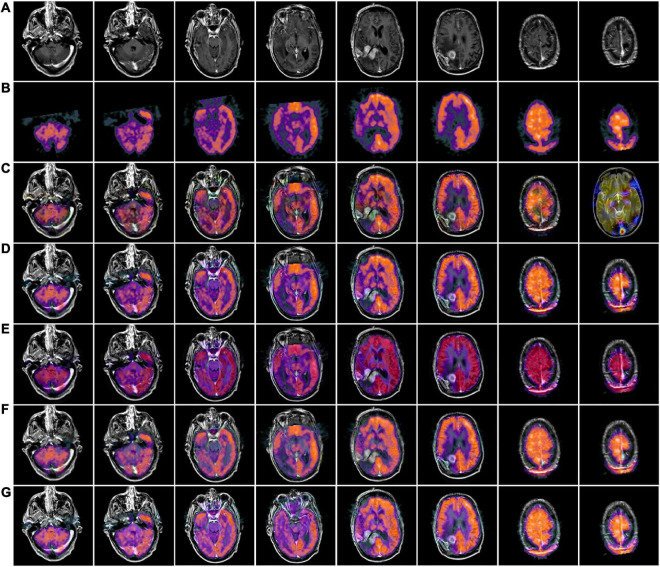
MRI-PET fusion images obtained by five methods in Paras1. **(A)** Magnetic resonance imaging (MRI) source image; **(B)** positron emission tomography (PET) source image; **(C)** nonsubsampled contourlet (NSCT) fusion result; **(D)** non-subsampled shear wave transform (NSST) fusion result; **(E)** guided filter ng fusion (GFF) fusion result; **(F)** Laplacian redecomposition (ReLP) fusion result; **(G)** fusion result of the proposed method.

**FIGURE 5:**
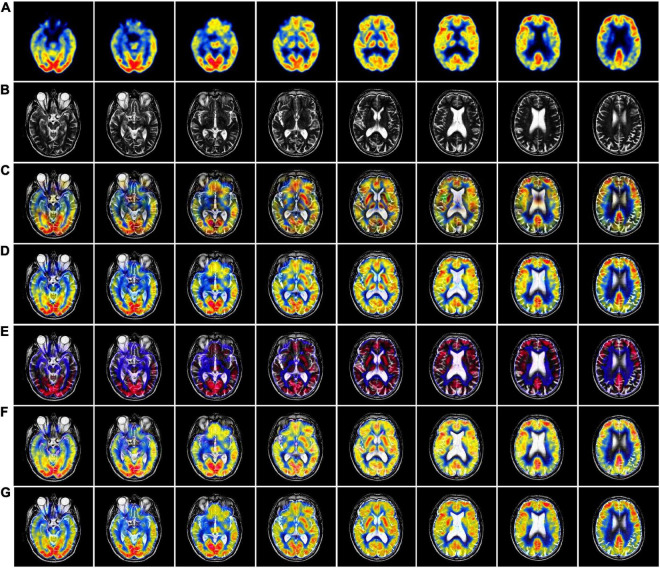
MRI-PET fusion images of five methods in Paras2. **(A)** Positron emission tomography (PET) source image; **(B)** magnetic resonance imaging (MRI) source image; **(C)** nonsubsampled contourlet (NSCT) fusion result; **(D)** non-subsampled shear wave transform (NSST) fusion result; **(E)** guided filter ng fusion (GFF) fusion result; **(F)** Laplacian redecomposition (ReLP) fusion result; **(G)** is the fusion result of the proposed method.

Figures 4, 5 use 42 sets of MRI-PET images from MRI-PET datasets Paras1 and Paras2, respectively. We randomly selected eight fused images. From Figure 4, it can be seen that NSCT and GFF show severe distortion, and the fused images of NSST and ReLP algorithms are too dark and have loss of detail information. In Figure 5, NSCT and NSST have dark luminance and GFF has distortion, while ReLP and our fusion algorithm have better visual effect.

In Table 2, by comparing 42 sets of fused images on the MRI-PET dataset, our proposed algorithm has the best mean value in objective evaluation metrics. Higher contrast, sharper edges and finer details were obtained. The subjective results of the fused images of the two algorithms, NSCT and GFF, were not satisfactory. NSCT and GFF had more color distortion. NSST showed abnormal brightness. ReLP performed better and was close to our average value. So far, it is easy to see that the multi-objective evaluation index of the integrated information is consistent with the conclusions of the subjective analysis. Our proposed algorithm significantly outperforms the average of all algorithms. In summary, we have a more comprehensive advantage over existing algorithms in the evaluation of objective metrics.

**TABLE 2:** Average values of index evaluation of different fusion methods for MRI-PET.

CT-MRI	Methods	MI	SF	AG	EI	NIQE	TMQI
Paras1	NSCT	3.7269	15.2214	6.1476	45.3264	4.5158	0.8123
	NSST	3.8261	15.3842	6.1842	47.8139	4.5927	0.8546
	GFF	4.1027	16.7253	6.0878	46.9367	4.5231	0.7712
	ReLP	4.1503	17.2636	6.2913	48.4811	4.6080	0.8582
	Proposed	4.2018	17.3059	6.3682	50.6531	4.6675	0.8654
Paras2	NSCT	3.6535	16.1356	6.0631	46.6235	4.6825	0.7403
	NSST	3.7282	16.8575	6.0051	45.3315	4.7057	0.6790
	GFF	3.7451	17.2243	6.2527	46.8728	4.6817	0.7052
	ReLP	3.7871	17.7991	6.3517	46.3297	4.7354	0.7106
	Proposed	3.8010	18.1052	6.4063	47.2031	4.7521	0.7149

### 3.4. MRI-SPECT fusion results comparison

**FIGURE 6:**
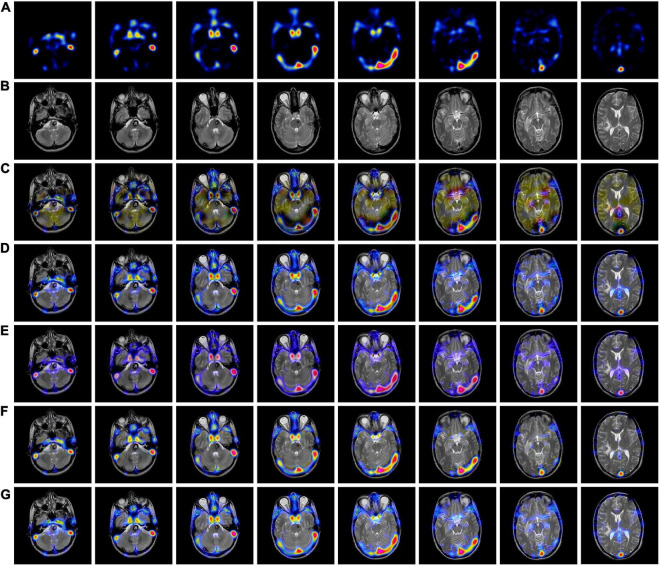
MRI-SPECT fusion images of the five methods in Paras1. **(A)** Single photon emission computed tomography (SPECT) source image; **(B)** magnetic resonance imaging (MRI) source image; **(C)** nonsubsampled contourlet (NSCT) fusion result; **(D)** non-subsampled shear wave transform (NSST) fusion result; **(E)** guided filter ng fusion (GFF) fusion result; **(F)** Laplacian redecomposition (ReLP) fusion result; **(G)** fusion result of the proposed method.

**FIGURE 7:**
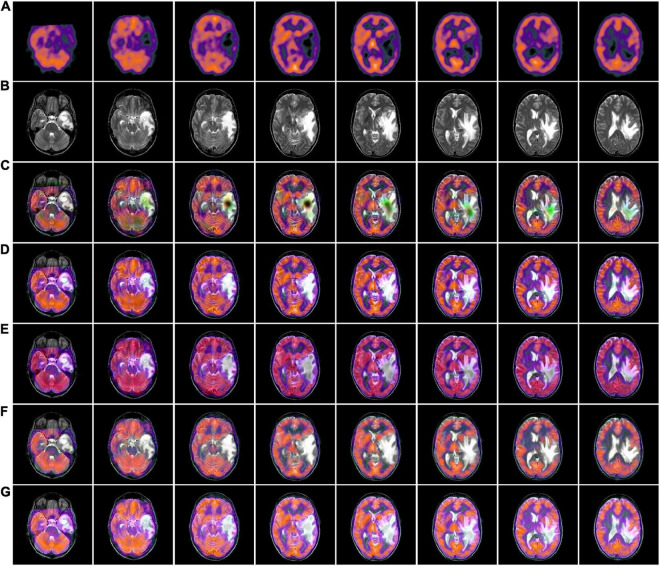
MRI-SPECT fusion images of the five methods in Paras2. **(A)** Single photon emission computed tomography (SPECT) source image; **(B)** magnetic resonance imaging (MRI) source image; **(C)** nonsubsampled contourlet (NSCT) fusion result; **(D)** non-subsampled shear wave transform (NSST) fusion result; **(E)** guided filter ng fusion (GFF) fusion result; **(F)** Laplacian redecomposition (ReLP) fusion result; **(G)** fusion result of the proposed method.

Figures 6, 7 we used MRI-SPECT datasets Paras1 and Paras2, respectively 52 sets of AD MRI-PET image fusion images for comparison. It can be seen from the figures that NSCT shows severe distortion, NSST fused images are too dark, GFF shows brightness anomalies, and ReLP does not perform well in terms of detail texture. Our fusion algorithm performs best in brightness, detail texture and edge contour.

In Table 3, we use 52 sets of fused images on the MRI-SPECT dataset for comparison, and our proposed DCT multiscale decomposition obtains sharper edges and finer details. The improved NMSF fusion rule obtains better brightness and contrast. The superiority of our method over other algorithms is demonstrated.

**TABLE 3:** Average values of index evaluation of different fusion methods for MRI-SPECT.

MRI-SPECT	Methods	MI	SF	AG	EI	NIQE	TMQI
Paras1	NSCT	3.6302	16.4106	6.2750	46.6732	4.2038	0.7931
	NSST	3.6231	15.8942	6.1773	47.0792	4.3107	0.7785
	GFF	3.7047	16.5378	6.2035	47.6470	4.2619	0.8063
	ReLP	3.8503	17.0346	6.3106	48.5718	4.3250	0.8307
	Proposed	3.8826	17.2069	6.4209	49.5014	4.4030	0.8526
Paras2	NSCT	3.6057	15.7431	6.1821	47.1003	4.3717	0.7352
	NSST	3.6183	15.7215	6.1040	46.6993	4.4602	0.7075
	GFF	3.6903	15.4903	6.2183	47.2854	4.3736	0.7202
	ReLP	3.7681	16.3107	6.3608	48.5810	4.4071	0.7464
	Proposed	3.7916	16.5012	6.4112	48.7106	4.5306	0.7503

**FIGURE 8:**
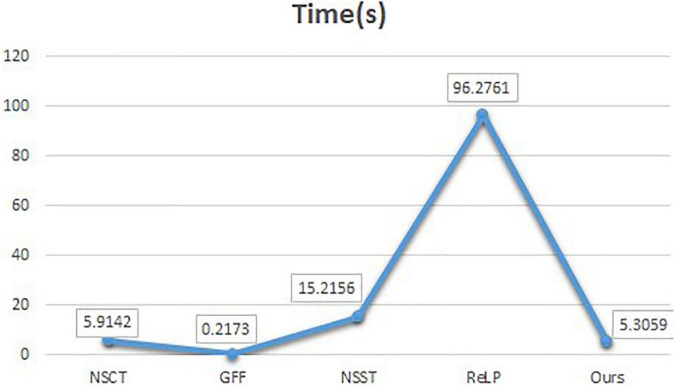
Comparison of running time.

To compare the advantages of the proposed methods more comprehensively, we calculate the running times of the comparison methods on the same pair of images of 256×256 size. Figure 8 shows the line graphs of the average running times of our method and the four comparison methods. The ReLP method has the longest running time and GFF has the shortest running time. From the line graph, it can be seen that our fusion method has the second best running speed than most of the other algorithms. However, medical image fusion is used to assist in diagnosis and treatment, and the effectiveness of the proposed method is demonstrated from objective and subjective evaluations. Therefore, the proposed method guarantees the quality of fusion results within an acceptable time consumption.

### 3.5. Comparison of subjective evaluation

Computed tomography (CT) or MRI unimodal imaging can no longer meet the demand for precise diagnosis in neurosurgery. A multimodality imaging technique that can clearly, visually, and holistically show AD brain atrophy and its association with surrounding cerebral vessels, nerves, and brain tissues can only accommodate the development of neurosurgical precision surgery. Quality assessment of multimodal fusion requires additional medical expertise. Therefore, we invited six chief neurosurgeons with more than 5 years of experience, and we randomly selected a test sample of 10 groups, each group including five fusion images. The subjective evaluation criteria were double stimulus continuous quality scale (DSCQS) including contrast, detail and invariance and acceptability scores of [1 (worst) to 5 (best)]. 1 indicates a non-diagnostic image and 5 indicates a good quality diagnostic image. Pathological invariance was scored as 0 (change) or 1 (no change). Table 4 shows the ratings of six clinicians, and the optimal values are shown in bold.

**TABLE 4:** Subjective quality evaluation of different fusion algorithms.

Method	CT-MRI				MRI-SPECT			
	**Contrast ratio↑**	**Detail↑**	**Invariance↑**	**Acceptability↑**	**Contrast ratio↑**	**Detail↑**	**Invariance↑**	**Acceptability↑**
NSCT	3.8 ± 0.52	3.6 ± 0.25	0.5 ± 0.39	3.6 ± 0.97	3.7 ± 0.85	3.8 ± 0.66	0.6 ± 0.14	3.7 ± 0.72
NSST	3.7 ± 0.68	3.7 ± 0.13	0.6 ± 0.37	3.5 ± 0.55	3.7 ± 0.63	3.9 ± 0.45	0.5 ± 0.97	3.6 ± 0.26
GFF	3.9 ± 0.43	3.6 ± 0.07	0.5 ± 0.93	3.7 ± 0.70	3.9 ± 0.51	4.0 ± 0.23	0.7 ± 0.41	3.7 ± 0.91
ReLP	4.0 ± 0.24	3.8 ± 0.64	0.7 ± 0.86	3.8 ± 0.61	4.1 ± 0.07	4.1 ± 0.58	0.7 ± 0.80	4.0 ± 0.96
Proposed	4.1 ± 0.19	3.9 ± 0.40	0.8 ± 0.75	4.0 ± 0.63	4.1 ± 0.79	4.2 ± 0.11	0.8 ± 0.05	4.1 ± 0.18

In Table 4, the subjective physician evaluations of CT-MRI and MRI-SPECT fusion are presented. The NSCT and GFF contrast and brightness were insufficient and therefore rated low. The GFF showed the worst distortion acceptability evaluation. The ReLP was very close to our evaluation among the four evaluation metrics. Our algorithm has the best performance in edge detail, luminance, contrast and spatial coherence, and received the best physician evaluation.

## 4. Conclusion

Multimodal neuroimaging data have high dimensionality and complexity, and seeking efficient methods to extract valuable features in complex datasets is the focus of current research. To address the shortcomings of AD multimodal fusion images such as contrast reduction, detail blurring and color distortion, we propose a multimodal fusion algorithm for Alzheimer’s disease based on DCT convolutional SR. The DCT multi-scale decomposition of the source medical image is performed to obtain the basic layer, local average energy layer and texture layer of the input image, and then the sub-images of different scales are used as training images respectively. The sub-dictionaries at different scales are optimally solved using the ADMM algorithm, and then convolutional sparse coding is performed, and the inverse DCT transform of the subimage coefficients is performed using a combination of improved L_1_ parameters and improved NMSF rules to obtain the multimodal fusion images. We experimentally demonstrate that the algorithm has sharper edge details, better color and spatial consistency than other algorithms by fusing medical images in three modalities, CT-MRI, MRI-PET, and MRI-SPECT. This proves that our algorithm outperforms existing state-of-the-art algorithms. In the future, we will use deep learning models for medical image multimodality classification and prediction, and apply them to early clinical diagnosis of AD.

## Data availability statement

The original contributions presented in this study are included in the article/supplementary material, further inquiries can be directed to the corresponding author/s.

## Ethics statement

Ethical review and approval was not required for the study on human participants in accordance with the local legislation and institutional requirements. Written informed consent for participation was not required for this study in accordance with the national legislation and the institutional requirements.

## Author contributions

GZ and XN: investigation, methodology, software, validation, visualization, and writing—original draft. BL, JL, and HY: investigation, methodology, software, and supervision. WS and SH: conceptualization, data curation, formal analysis, funding acquisition, methodology, project administration, resources, supervision, validation, and writing—review and editing.
